# A Case of Laparoscopic Resection for Carcinoma of the Gastric Remnant following Proximal Gastrectomy Reconstructed with Jejunal Interposition

**DOI:** 10.1155/2016/9357659

**Published:** 2016-03-13

**Authors:** Kazuhito Yajima, Yoshiaki Iwasaki, Ken Yuu, Ryouki Oohinata, Misato Amaki, Yoshinori Kohira, Souichiro Natsume, Satoshi Ishiyama, Keiichi Takahashi

**Affiliations:** Department of Surgery, Tokyo Metropolitan Cancer and Infectious Diseases Center, Komagome Hospital, 3-18-22 Honkomagome, Bunkyo-ku, Tokyo 113-8677, Japan

## Abstract

A 72-year-old Japanese man had a history of proximal gastrectomy for early gastric cancer located in the upper third of the stomach in 2007. Our usual treatment strategy for early gastric cancer in the upper third of the stomach in 2007 was open proximal gastrectomy reconstructing by jejunal interposition with a 10 cm single loop. Upper gastrointestinal fiberscopy for annual follow-up revealed a type 0-IIc-shaped tumor with ulcer scar, 4.0 cm in size, located in the gastric remnant near the jejunogastrostomy. A clinical diagnosis of cancer of the gastric remnant, clinical T1b(SM)N0M0, Stage IA, following the proximal gastrectomy was made and a laparoscopic approach was selected because of the cancer's early stage. Remnant total gastrectomy with D1 plus lymphadenectomy was carried out with five ports by a pneumoperitoneal method. Complete resection of the reconstructed jejunum was undergone along with the jejunal mesentery. Reconstruction by the Roux-en-Y method via the antecolic route was selected. Total operative time was 395 min and blood loss was 40 mL. Our patient was the first successful case of resection for carcinoma of the gastric remnant following proximal gastrectomy reconstructed with jejunal interposition in a laparoscopic approach.

## 1. Introduction

Laparoscopic gastrectomy has become widely used for the treatment of early gastric cancer in Japan; the feasibility of laparoscopic distal gastrectomy in particular has been assessed in several studies [1–3]. However, laparoscopic procedures are more challenging in patients with previous abdominal surgery because of a higher risk of enteric injury, technical difficulties associated with adhesions, and longer operative times. In terms of the recent advantages of the laparoscopic technique, several reports have presented the usefulness of the laparoscopic approach for patients with remnant gastric cancer following radical distal gastrectomy [4–12]. We here present a case of laparoscopic resection for carcinoma of the gastric remnant following proximal gastrectomy reconstructed with jejunal interposition.

## 2. Case Presentation

**Figure 1:**
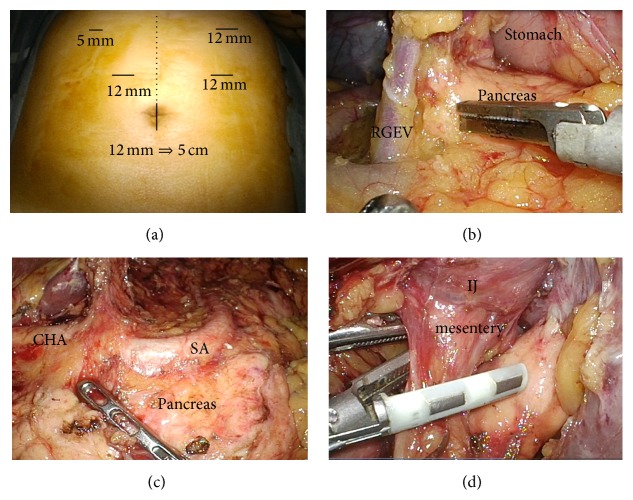
Port sites and lymphadenectomy at laparoscopic remnant total gastrectomy. (a) The port sites and scar of initial gastrectomy (dotes line). (b) Station number 6 lymph nodes were completely removed (RGEV; right gastroepiploic vein). (c) The suprapancreatic lymph nodes (station numbers 8a, 9, and 11p) were dissected* en bloc* (CHA; common hepatic artery, SA; splenic artery). (d) The mesentery of the interposed jejunum (IJ) was transected using a vessel sealing system (LigaSure*™* Blunt Tip 5 mm–37 mm, Covidien, Tokyo, Japan).

A 72-year-old Japanese man had a history of proximal gastrectomy for early gastric cancer located in the upper third of the stomach in 2007. Our usual treatment strategy for early gastric cancer in the upper third of the stomach at this time was open proximal gastrectomy reconstructing by jejunal interposition with a 10 cm single loop. In this patient, interposed jejunum was approached via the retrocolic route. Lymphadenectomy was D1 (station numbers 1, 2, 3a, 4sa, 4sb, and 7) according to the Japanese Gastric Cancer Association (JGCA) guidelines for initial proximal gastrectomy [13]. Upon physical examination, he was found to be 165 cm in height and weighed 55 kg. There was an upper middle operative scar, 17 cm in length, in his abdomen. All of the laboratory data were within the normal range and the tumor markers CEA and CA 19-9 were 7.4 ng/mL and 9.7 U/mL, respectively. Upper gastrointestinal fiberscopy for annual follow-up revealed a type 0-IIc-shaped tumor with ulcer scar, 4.0 cm in size, located in the gastric remnant near the jejunogastrostomy (Figure 1). Biopsy specimen showed well-differentiated adenocarcinoma. A clinical diagnosis of early gastric cancer according to the Japanese Classification of Gastric Carcinoma [14], T1b(SM)N0M0, Stage IA, following the proximal gastrectomy was made. A laparoscopic approach was selected for remnant gastrectomy because of the cancer's early stage.

**Figure 2:**
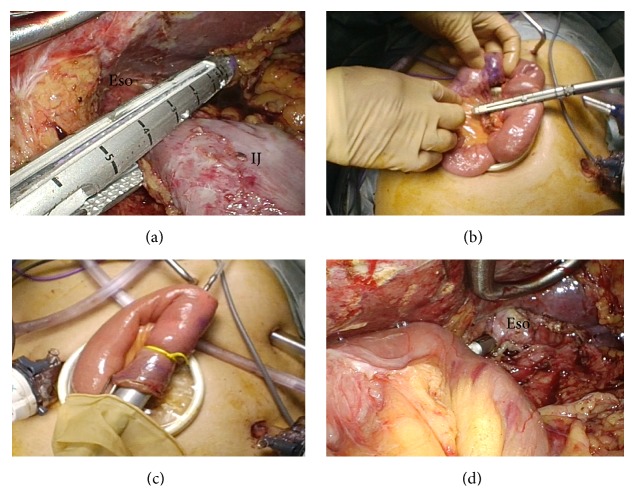
Reconstruction at laparoscopic remnant total gastrectomy. (a) The esophagus was transected using a linear stapler (GIA Tri-Staple 60 mm, purple 60 mm, Covidien) on the proximal side of the anastomosis of the esophagojejunostomy at initial proximal gastrectomy. (b) Jejunojejunostomy was carried out by side-to-side anastomosis using a linear stapler extracorporeally. (c, d) Esophagojejunostomy was performed by an end-to-side anastomosis using a circular stapler (DST Series EEA Staplers, 25 mm, Covidien) intracorporeally (IJ; interposes jejunum, Eso; esophagus).

Surgery was carried out with five ports by the pneumoperitoneal method using our usual laparoscopic technique (Figure 1(a)). The lateral segment of the liver was retracted using a Nathanson Liver Retractor (Cook Surgical, Bloomington, Indianapolis, USA). At initial laparoscopy, there was a little adhesion in the median abdominal incision. At first, adhesion was removed as much as possible and omentum was dissected to open the bursa. The tightest adhesion in this operation was that between the lateral segment of the liver and the dissected lesser curvature of the remnant stomach. The right gastroepiploic artery and vein were clipped and divided, and station number 6 lymph nodes were completely removed (Figure 1(b)). After dividing the right gastric artery (station number 5), the duodenum was transected using a linear stapler (GIA*™* Tri-Staple*™* 60 mm, purple 60 mm, Covidien, Tokyo, Japan). The suprapancreatic lymph nodes (station numbers 8a, 9, and 11p) could be easily dissected* en bloc* because these regions were not dissected at initial proximal gastrectomy (Figure 1(c)). After gastrectomy, the reconstructed jejunum was resected. The mesentery of the interposed jejunum was transected using a vessel sealing system (LigaSure*™* Blunt Tip 5 mm–37 mm, Covidien) (Figure 1(d)). After the abdominal esophagus had been scarified circumferentially, the esophagus was transected using a linear stapler on the proximal side of the anastomosis of the esophagojejunostomy at initial proximal gastrectomy (Figure 2(a)). The remnant stomach and interposed jejunum were successfully reduced back into the peritoneal cavity through the umbilical port-side. A minilaparotomy incision of about 5 cm was made in the umbilical port site, for an Alexis® Wound Retractor S (Applied Medical Resources Co., Tokyo, Japan). Reconstruction using the Roux-en-Y method via the antecolic route was performed. At first, jejunojejunostomy was carried out by side-to-side anastomosis using a linear stapler extracorporeally (Figure 2(b)). Next, esophagojejunostomy was performed by an end-to-side anastomosis using a circular stapler (DST Series*™* EEA*™* Staplers, 25 mm, Covidien) (Figures 2(c) and 2(d)). Total operative time was 395 min and blood loss was 40 mL.

**Figure 3:**
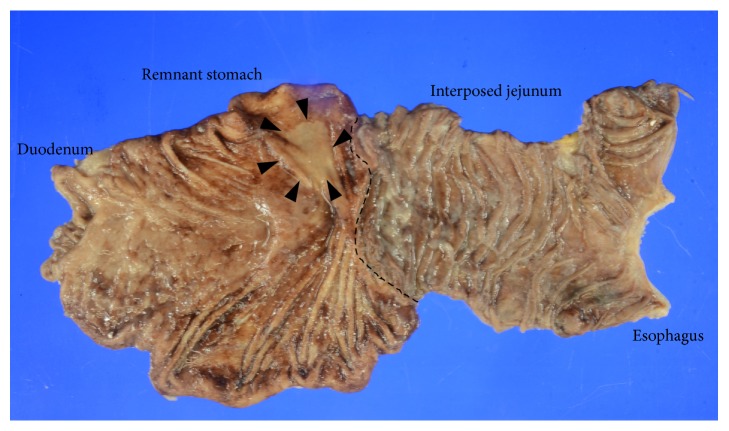
Resected specimen. The resected stomach contained a superficial depressed-type tumor, 37 × 17 mm in size (black arrowhead). The black dote line showed the jejunogastrostomy at initial gastrectomy.

The postoperative course was uneventful and the patient was discharged on the 10th postoperative day. The resected stomach contained a superficial depressed-type tumor, 37 × 17 mm in size (Figure 3, black arrowhead). Histological examination revealed well-differentiated adenocarcinoma to the depth of the mucosa, with no lymph node metastasis, which was pathologically classified as Stage IA.

## 3. Discussion

We have described a 72-year-old Japanese man who underwent a laparoscopic approach for carcinoma of the gastric remnant following proximal gastrectomy reconstructed with jejunal interposition. To the best of our knowledge, this is the first reported case of laparoscopic resection for remnant stomach reconstructed by jejunal interposition in the English language literature. In our case, the remnant total gastrectomy with* en bloc* lymphadenectomy could be completed easily and safely using a laparoscopic approach.

Remnant gastric cancer was originally defined as gastric cancer arising after distal gastrectomy for benign disease. More recently, remnant gastric cancer has been used to refer to all cancers arising in the remnant stomach, regardless of initial disease or operation. According to previous reports, with proximal gastrectomy becoming a conventional type of gastrectomy, the incidence of remnant cancer following proximal gastrectomy is increasing [15]. Ohyama et al. reported that gastric stump carcinoma following proximal gastrectomy occurred at a high frequency of 5.4% of initial resections and the time interval between the initial gastrectomy and the treatment of gastric stump cancer was within 5 years in 3 patients, within 5–10 years in 8, and after 10 years in 6. In our case, the occurrence of gastric remnant cancer was 7 years after the initial surgery.

Yamada et al. [4] first reported a laparoscopy-assisted complete gastrectomy for patients with early gastric remnant cancer in 2005. Since then, there have been increasing reports of successful laparoscopy-assisted gastrectomy for gastric remnant cancer. To date, just a hundred patients have undergone it by a laparoscopic approach for remnant gastric cancer [12]. Among these, laparoscopic total gastrectomy following proximal gastrectomy was reported in only three cases [8, 9, 12]. Proximal gastrectomy is performed widely as a function-preserving operation for early gastric cancer located in the upper third of the stomach. In terms of reconstruction after proximal gastrectomy, three reconstruction methods are mainly used: esophagogastrostomy [16, 17], jejunal interposition [18–20], and double-tract method [21]. To avoid reflux esophagitis after proximal gastrectomy, jejunal interposition has been selected for the reconstruction method in our department since 1973 and, to date, more than 200 cases have undergone this surgical procedure [22]. All of the three reported cases [8, 9, 12] were initial reconstruction of esophagogastrostomy; this is the first reported case of laparoscopic resection for remnant stomach reconstructed by jejunal interposition in the English language literature.

Cases of resection for remnant stomach reconstructed by jejunal interposition have been rarely reported, even in open surgery. Nozaki et al. reported five cases with removal of remnant gastric cancer following proximal gastrectomy with jejunal interposition [23]. The unique technique of Nozaki et al. was preserving the interposed jejunum for re-reconstruction by Roux-en-Y anastomosis. In our case, removal of the total gastric remnant with radical lymphadenectomy was the same as that of Nozaki et al. On the other hand, the interposed jejunum was totally removed at the same time. In our case, the first reason for the total removal of interposed jejunum was the difficulty of safely preserving the vessels feeding the interposed jejunum owing to severe adhesions. The second reason for the total removal of interposed jejunum was that esophagojejunostomy was a usual and safe anastomotic technique after laparoscopy-assisted total gastrectomy or proximal gastrectomy. Accordingly, we selected total removal of the gastric remnant with interposed jejunum and re-reconstruction by the Roux-en-Y method.

The selected surgical approach after major surgery for intra-abdominal malignancies has usually been the conventional open method. Kwon et al. [10] and Son et al. [9] reported a comparison of the surgical outcomes between an open approach group and a minimally invasive approach group for patients with remnant gastric cancer. In their report, compared with the open approach group, the minimally invasive approach group for remnant gastric cancer demonstrated better short-term outcome and comparable oncologic results. On the other hand, eight of 17 patients (47.1%) required conversion to open surgery because of the presence of severe intra-abdominal adhesions [9]. Adhesion formation is one of the major concerns in patients who undergo major abdominal surgery. In laparoscopic surgery after major abdominal surgery, the first key to a safe procedure is the insertion of the first trocar in an area free from adhesion. In our case, the first trocar was inserted from the left lower oblique to an open method. The second key was careful lysis of adhesion from the abdominal wall. If all trocars are inserted at the same site of laparoscopic gastrectomy, removal of remnant stomach, and lymphadenectomy, reconstruction might be performed by the usual method easily.

## 4. Conclusion

We have described a good result of a laparoscopic approach for early gastric remnant cancer following radical proximal gastrectomy. A review of the literature supports a minimally invasive approach for this procedure, showing that it is safe, effective, and technically feasible.
